# m6A Regulator-Mediated Tumour Infiltration and Methylation Modification in Cervical Cancer Microenvironment

**DOI:** 10.3389/fimmu.2022.888650

**Published:** 2022-04-29

**Authors:** Wenyi Zhang, Pei Xiao, Jiayi Tang, Rui Wang, Xiangdong Wang, Fengxu Wang, Junpu Ruan, Shali Yu, Juan Tang, Rongrong Huang, Xinyuan Zhao

**Affiliations:** ^1^ Department of Occupational Medicine and Environmental Toxicology, Nantong Key Laboratory of Environmental Toxicology, School of Public Health, Nantong University, Nantong, China; ^2^ Center for Non-Communicable Disease Management, Beijing Children’s Hospital, Capital Medical University, National Center for Children’s Health, Beijing, China; ^3^ Department of Pharmacy, Affiliated Hospital of Nantong University, Nantong, China

**Keywords:** m6A regulators, tumour microenvironment, immunotherapy, prognosis, cervical cancer

## Abstract

**Background:**

N6-methyladenosine (m6A) is the most abundant internal mRNA modification in eukaryotic cells. There is accumulating evidence that m6A methylation can play a significant role in the early diagnosis and treatment of cancers. However, the mechanism underlying the involvement of m6A in cervical cancer remains unclear.

**Methods:**

Here, we examined the m6A modification patterns of immune cells in the tumour microenvironments (TMEs) of 306 patients with cervical cancer from The Cancer Genome Atlas dataset and analysed the relations between them according to 32 m6A regulators. Immune infiltration in the TME of cervical cancer was analysed using the CIBERSORT algorithm and single-sample gene set enrichment analysis. The m6Ascore was structured though principal component analysis.

**Results:**

Two different m6A modification patterns were detected in 306 patients with cervical cancer, designated as m6Acluster A and B. The immune cell infiltration characteristics and biological behaviour differed between the two patterns, with m6Acluster A showing a higher level of immune infiltration. The samples were also divided into two genomic subtypes according to 114 m6A regulatory genes shown to be closely correlated with prognosis on univariate Cox regression analysis. Survival analysis showed that gene cluster B was related to better survival than gene cluster A. Most of the m6A regulators showed higher expression in gene cluster B than in gene cluster A. Single-sample gene set enrichment analysis indicated a higher level of immune cell infiltration in gene cluster A. The m6Ascore signature was examined to determine the m6A modification patterns in cervical cancer. Patients with a high m6Ascore showed better survival, while the low m6Ascore group had a higher mutation frequency and better response to treatment.

**Conclusions:**

This study showed that m6A modification patterns play important roles in cervical cancer. Analysis of m6A modification patterns will yield an improved understanding of the TME in cervical cancer, and facilitate the development of better immunotherapy strategies.

## Introduction

Cervical cancer is the fourth most common malignant tumour in women worldwide and a major cause of morbidity and mortality in low-income countries (1). There were an estimated 570,000 new cases of cervical cancer, accounting for 3.15% of all cancer cases, and approximately 40,000 deaths, accounting for 3.26% of all cancer-related deaths worldwide in 2018 (2). These figures show the importance of accurate prognostic predictors for patients with cervical squamous cell carcinoma (CESC) to allow personalised treatment.

N6-methyladenosine (m6A) is the most common internal modification on eukaryotic mRNA. Regulation of m6A is mediated by ‘writers’ that catalyse its addition *via* the methyltransferase complex, ‘erasers’ responsible for its removal *via* the action of demethylase and ‘readers’ consisting of RNA-binding proteins that are responsible for its recognition (3). Here, we examined 32 regulators, consisting of 10 writers (*METTL3*, *METL14*, *METL16*, *WTAP*, *WTAP*, *ZC3H13*, *CBLL1*, *RBM15*, *NSUN2* and *RBM15B*), 20 readers (*YTHDC1*, *YTHDC2*, *YTHDF1*, *YTHDF2*, *YTHDF3*, *HNRNPC*, *FMR1*, *LRPPRC*, *HNRNPA2B1*, *IGFBP1*, *IGFBP2*, *IGFBP3*, *FXR1*, *EIF4G2*, *EIF3A*, *ABCF1*, *G3BP1*, *ELAVL1*, *FXR2* and *RBMX*) and two erasers (*FTO* and *ALKBH5*). Studies have shown that m6A regulators play significant roles in the progression of cancer, including proliferation, migration and invasion (4). However, further research is needed to determine the therapeutic utility of m6A regulators and their effects on prognosis in CESC.

The tumour microenvironment (TME) has broad implications for tumorigenesis because it can affect the development and progression of cancer through the circulatory and lymphatic systems by harbouring tumour cells that interact with surrounding cells (5). T cells and mast cells have been suggested to be significantly associated with the survival rate of patients with cervical cancer (6). Human papillomavirus (HPV) infection and its oncoproteins help cervical tumour cells to evade attack by natural killer (NK) cells, while NK cells can recognise and eliminate cervical tumour cells and cells infected by the virus (7).

Targeting of the checkpoints of immune cell activation, such as cytotoxic T lymphocyte-associated protein 4 (CTLA-4) and programmed cell death protein 1 (PD-1), have been shown to be effective means of activating anti-tumour immune responses (8). A number of studies have shown that the ligand of PD-1, programmed death ligand 1 (PD-L1), and CTLA-4 are powerful prognostic factors as well as therapeutic targets in cervical cancer (9, 10). The key in discussing new immunisation strategies and studying the reactions to existing immune checkpoint inhibitors is the prediction of immune infiltration based upon the characteristics of the cells in the TME (11). We can improve the abilities to guide and predict the response to immunotherapy by comprehensively analysing the heterogeneity and complexity of the TME landscape. This will also aid in the search for new therapeutic targets (12). A number of recent studies have shown that there are close relations between TME-infiltrating immune cells and m6A modification. Lack of the m6A reader *YTHDF2* can disrupt the anti-tumour and anti-viral activities of NK cells. Meanwhile, *YTHDF2* has been shown to modulate the transport of NK cells and regulate expression of the transcription factor eomesodermin to maintain the terminal differentiation and homeostasis of NK cells (13). Defects in the reader *YTHDF1* were accompanied by a high anti-tumour response of antigen-specific CD8^+^ T cells. Mechanistically, dendritic cells lacking *YTHDF1* failed to identify transcripts encoding lysosomal proteases, thereby reducing the expression of lysosomal cathepsins (14).

Studies have stressed the relations between single m6A regulators and cell type. Here, we comprehensively analysed the TME cell infiltration characterisations mediated by multiple m6A regulators based on clinical information and transcriptome data of 306 CESC patients from the Cancer Genome Atlas (TCGA) dataset. According to the expression pattern of m6A regulators, consensus clustering was performed by principal component analysis (PCA), survival analysis and single-sample gene set enrichment analysis (ssGSEA). Then, we constructed m6Ascore signatures and explored their relations to the immune microenvironment and response to immunotherapy.

## Methods

### Sources of Cervical Cancer Data

Gene expression data and clinical annotations of the datasets on cervical cancer were retrieved from The Cancer Genome Atlas (https://portal.gdc.cancer.gov/), including 3 normal and 306 tumour samples. Samples with missing information were deleted for further study. To deal with the TCGA-CESC cohort, the TCGAbiolinks package by R software was used to transform RNA sequencing data (fragments per kilobase of transcript per million mapped reads [FPKM] values) (15). We then transformed FPKM values into transcripts per kilobase million (TPM) values. Controlling batch effect results from nonbiological technical biases by the R package “SVR” (16). The data of somatic mutations and copy number variants (CNV) in CESC were obtained from the database of the TCGA and visualized by R packages “ma*FTO*ols” and “Rcircos” (17, 18).

### Cluster Analysis of 32 m6A Regulators

32 m6A regulators: ten writers (METTL3, METL14, METL16, WTAP, WTAP, ZC3H13, CBLL1, RBM15, NSUN2 and RBM15B), twenty readers (YTHDC1, YTHDC2, YTHDF1, YTHDF2, YTHDF3, HNRNPC, FMR1, LRPPRC, HNRNPA2B1, IGFBP1, IGFBP2, IGFBP3, FXR1, EIF4G2, EIF3A, ABCF1, G3BP1, ELAVL1, FXR2, and RBMX), and two erasers (FTO and ALKBH5) were incorporated in the study. Univariate Cox analyses were adopted to evaluate the links between m6A regulators and prognosis of cervical cancer. Further, we used the R package “Consensus Cluster Plus” to conduct an unsupervised cluster analysis according to the 32 m6A regulatory genes. According to the clustering effect, the clustering stability was better when k=2.

### Functional Annotation and Gene Set Variation Analysis

The gene sets of “c2.cp.kegg.v7.5.symbols.gmt” and “c5.go.v7.5.symbols.gmt” were downloaded from the MSigDB database (19). In order to explore the variation of biological processes between the m6A types, we then used the R package “GSVA” to make GSVA enrichment analysis. We also conducted the functional annotation by the R package “clusterProfiler” for differentially expressed genes (DEGs) between m6A distinct phenotypes. The rank criterion was adjusted P-value < 0.05.

### Immune Cell Infiltration by ssGSEA

We carried out single-sample gene set enrichment analysis to explore the abundance of immune cells infiltrating the TME of CESC, including Activated B cell, Activated CD4 T cell, Activated CD8 T cell, Activated dendritic cell, CD56dim natural killer cell, Eosinophil, Immature B cell, MDSC, Mast cell, Monocyte, Natural killer T cell, Neutrophil and the like. The relative abundance of each TME infiltrating cell in each sample was expressed by the enrichment scores that were calculated by ssGSEA analysis.

### Analysis of Differentially Expressed Genes (DEGs) for the Determination of m6A Modification Patterns

According to two different m6A modification patterns that we have concluded, we screened out DEGs among CESC patients by the R package “limma.” The rank criterion was adjusted P-value < 0.001. Likewise, survival-related DEGs of the two different m6A modification patterns were screened out by the univariate Cox model and the R package “Consensus Cluster Plus” was used for making cluster analysis based upon the DEGs. Further, the survival of different genotypes and the differences in the expression of m6A regulators were compared.

### Construction of m6A Gene Signature

We then constructed an m6A scoring system based on the results that have been obtained. Next, principal components were analyzed by PCA analysis, and we chose the principal components 1 and 2 as feature scores. It is largely concentrated in the score of the gene block with the most significant correlation or inverse correlation. Meanwhile, it incorporated the influence of untracked genes to other members of the set into consideration scope. The m6A score was calculated by the formula: m6Ascore = Ʃ(PCli+PC2i). In the formula, “i” represents m6A phenotype-related genes (20).

### Genomic and Clinical Information of Immune Checkpoints

We used the Wilcoxon test to analyse differential expression of immune checkpoints between low and high m6A score groups, including CTLA4, PD-L1 and PD-1. Simultaneously, we downloaded the immune checkpoint inhibitor (ICI) immunophenoscore (IPS) file from The Cancer Immunome Atlas Database. Immunophenoscore (IPS), a good index measuring tumour immunogenicity, was used for estimating the immunotherapeutic significance of m6A gene signature.

### Statistical Analysis

R (version 4.0.4) was used to analyse all statistical analysis. The one-way ANOVA and the Kruskal–Wallis test were used to compare the data between the multiple groups. The “surv-cutpoint” function was used to divide the best cutoff score between low and high m6Ascore groups. The prognostic survival curve was constructed by the Kaplan–Meier method. We evaluated the mutation status of patients in low- and high-m6Ascore subtypes by waterfall function in MA*FTO*ols package. The threshold of p < 0.05 indicating the significance of correlation applies to all analyses.

## Results

### Landscape of the Genetic Variation of m6A Regulators in Cervical Cancer

**Figure 1:**
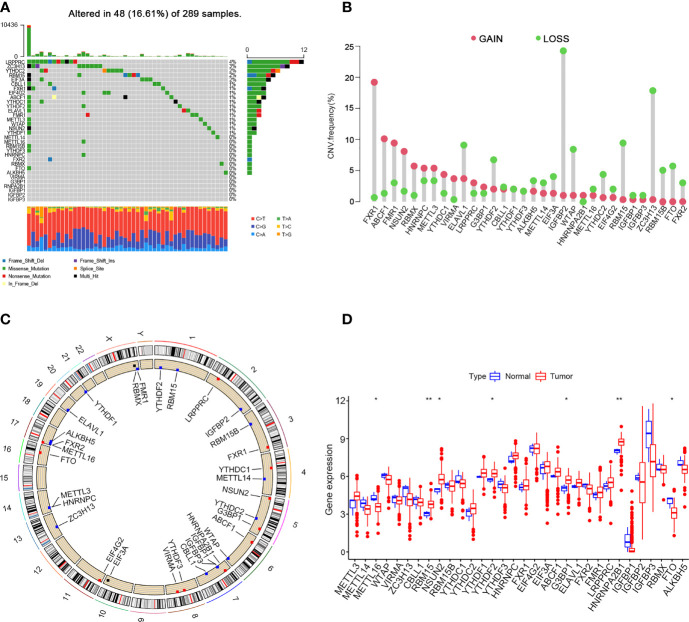
Landscape of genetic and expression variation of m6A regulators in cervical cancer. **(A)** 48 of 289 samples have genetic alterations of 32 m6A regulators. **(B)** Copy number variation mutation frequency of m6A regulators. **(C)** Position of copy number variation change of m6A regulators on human chromosome. **(D)** Difference in the expression level of m6A regulators between normal and tumour samples (*P < 0.05 and **P < 0.01).

In this study, 10 writers (METTL3, METL14, METL16, WTAP, WTAP, ZC3H13, CBLL1, RBM15, NSUN2 and RBM15B), 20 readers (YTHDC1, YTHDC2, YTHDF1, YTHDF2, YTHDF3, HNRNPC, FMR1, LRPPRC, HNRNPA2B1, IGFBP1, IGFBP2, IGFBP3, FXR1, EIF4G2, EIF3A, ABCF1, G3BP1, ELAVL1, FXR2 and RBMX) and two erasers (FTO and ALKBH5) were evaluated to explore the roles of 32 m6A regulatory genes in cervical cancer. First, copy number variations and somatic mutations in 32 m6A regulators in cervical cancer were studied (**Figure 1A**). Mutations in the m6A regulators were detected in 48 of 289 samples (16.61%). The LRPPRC gene showed the highest mutation rate (> 4%) followed by ZC3H13 (3%). FXR1, ABCF1, FMR1, NSUN2 and RBMX have higher frequencies of CNV amplification, while ELAVL1, YTHDF2, IGFBP2, WTAP, RBM15 and ZC3H13 have higher probabilities of CNV deletions (**Figure 1B**). **Figure 1C** shows the chromosomal positions with copy number variations of m6A regulators. In addition, to identify the relations between genetic variations and the expression of m6A regulators, we compared the mRNA expression levels of 32 m6A regulators between tumour and normal samples. The results suggested that the levels of METTL16 and FTO expression were reduced, while those of RBM15, NSUN2, YTHDF2, G3BP1 and HNRNPA2B1 were increased in tumour tissues (**Figure 1D**).

### Patterns of m6A Methylation Modification Mediated by 32 Regulators

**Figure 2:**
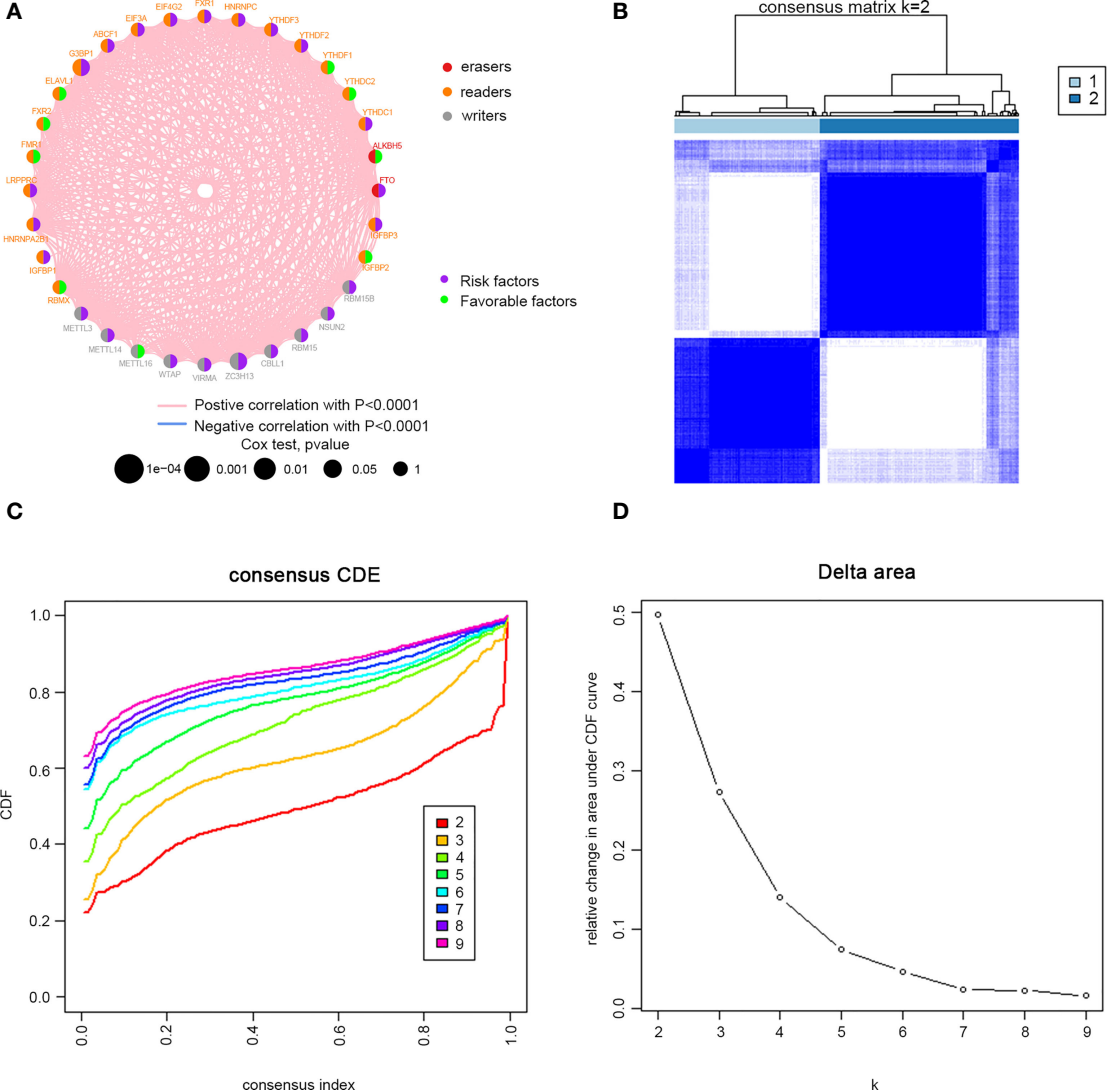
M6A methylation modification patterns in cervical cancer. **(A)** Interaction between m6A regulators. Red dots represent erasers, orange dots represent readers, and gray dots represent writers. Pink lines represent positive correlation between m6A regulators, and blue lines represent negative correlation between m6A regulators. The size of each circle represents the prognostic effect of each adjustment factor and is scaled by P value. Purple indicates risk factors, and green indicates favourable factors. Cervical cancer samples were classified to two m6Aclusters **(A** and **B)** according to the ConsensusClusterPlus algorithm. **(B)** Consensus matrix. **(C)** CDF graph. **(D)** Relative change of the area under the CDF curve when k = 2–9.

Clinical information was incorporated with the TCGA dataset. Univariate Cox regression analysis demonstrated the prognostic value of 32 m6A regulators in patients with cervical cancer (**Supplementary Table 1**). The m6A regulator network revealed the prognostic significance of m6A regulator interactions in patients with cervical cancer (**Figure 2A**). The expression of m6A regulators was strongly linked within the same functional category, as well as in erasers, readers and writers. We concluded that the relations between erasers, readers and writers and m6A modification patterns played important roles in the development and occurrence of cervical cancer. Then, we divided the TCGA cohort into two clusters, m6Acluster A (125 cases) and m6Acluster B (171 cases), based on the expression of m6A regulators (**Figures 2B, D**).

### Gene Set Variation Analysis and TME Cell Infiltration Characteristics in Different m6A Modification Modes

**Figure 3:**
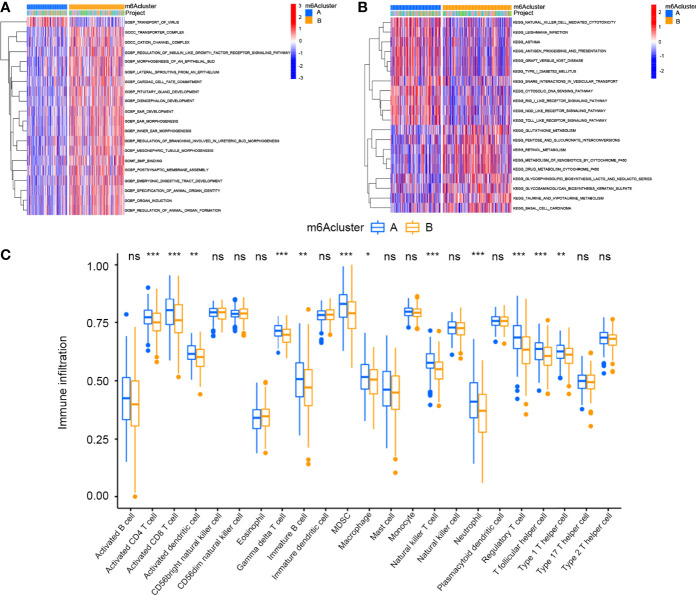
GSVA enrichment analysis and Tumor microenvironments cell infiltration characteristics in two m6A Modification Modes in cervical cancer. **(A)** GO enrichment analysis. **(B)** KEGG enrichment analysis. **(C)** Expression of immune-infiltrating cells in two m6A modification patterns (*P < 0.05, **P < 0.01, and ***P < 0.001). ns, not significant.

Gene set variation analysis (GSVA) was performed to investigate the biological behaviours of the two m6A modification clusters (**Figures 3A, B**). GO enrichment analysis showed that m6Acluster A was positively associated with transport of virus, while m6Acluster B was positively associated with transporter complex, cation channel complex, regulation of insulin-like growth factor receptor signalling pathway, morphogenesis of an epithelial bud, lateral sprouting from epithelium, cardiac cell fate commitment, pituitary gland development, diencephalon development, ear development, ear morphogenesis, inner ear morphogenesis, regulation of branching involved in ureteric bud morphogenesis, mesonephric tubule morphogenesis, bone morphogenic protein binding, postsynaptic membrane assembly, embryonic digestive tract development, specification of animal organ identity, organ induction and regulation of animal organ formation. KEGG enrichment analysis showed that m6Acluster A was positively associated with NK cell-mediated cytotoxicity, *Leishmania* infection, asthma, antigen processing and presentation, graft versus host disease, type I diabetes mellitus, snare interactions in vesicular transport, cytosolic DNA sensing pathway, rig I-like receptor signalling pathway, nod-like receptor signalling pathway and Toll-like receptor signalling pathway. By contrast, m6Acluster B was positively associated with glutathione metabolism, pentose and glucuronate interconversion, retinol metabolism, metabolism of xenobiotics by cytochrome p450, drug metabolism by cytochrome p450, glycosphingolipid biosynthesis lacto and neolacto series, glycosaminoglycan biosynthesis keratin sulphate, taurine and hypotaurine metabolism and basal cell carcinoma. Furthermore, we analysed TME cell infiltration of the different clusters. The results showed that m6Acluster A was significantly enriched in immune cells, including activated CD4^+^ T cells, activated CD8^+^ T cells, activated dendritic cells, γδT cells, immature B cells, myeloid-derived suppressor cells (MDSCs), macrophages, NK cells, neutrophils, regulatory T (Treg) cells, follicular helper T (Tfh) cells and type 1 helper T (Th1) cells (**Figure 3C**).

### Differentially Expressed Genes Between Distinct m6A Phenotypes

**Figure 4:**
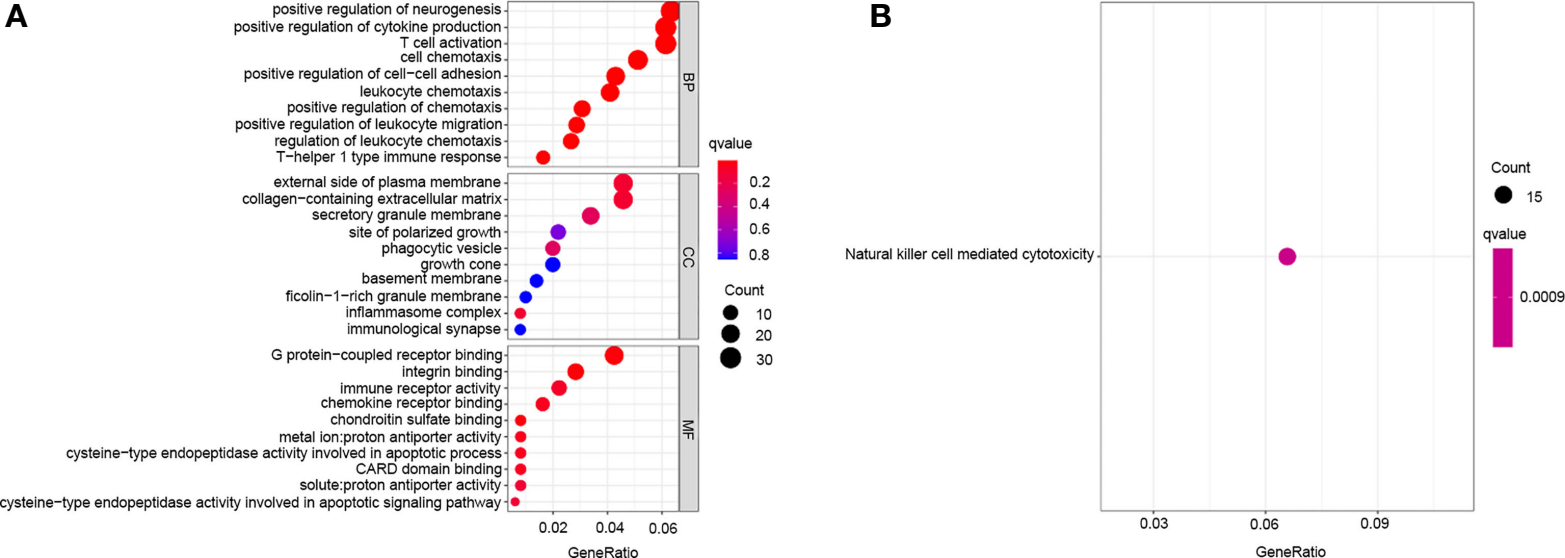
Transcriptome characteristics of m6A modification patterns in cervical cancer. **(A)** GO enrichment analysis of m6A differentially expressed genes. **(B)** KEGG pathways of m6A differentially expressed genes.

**Figure 5:**
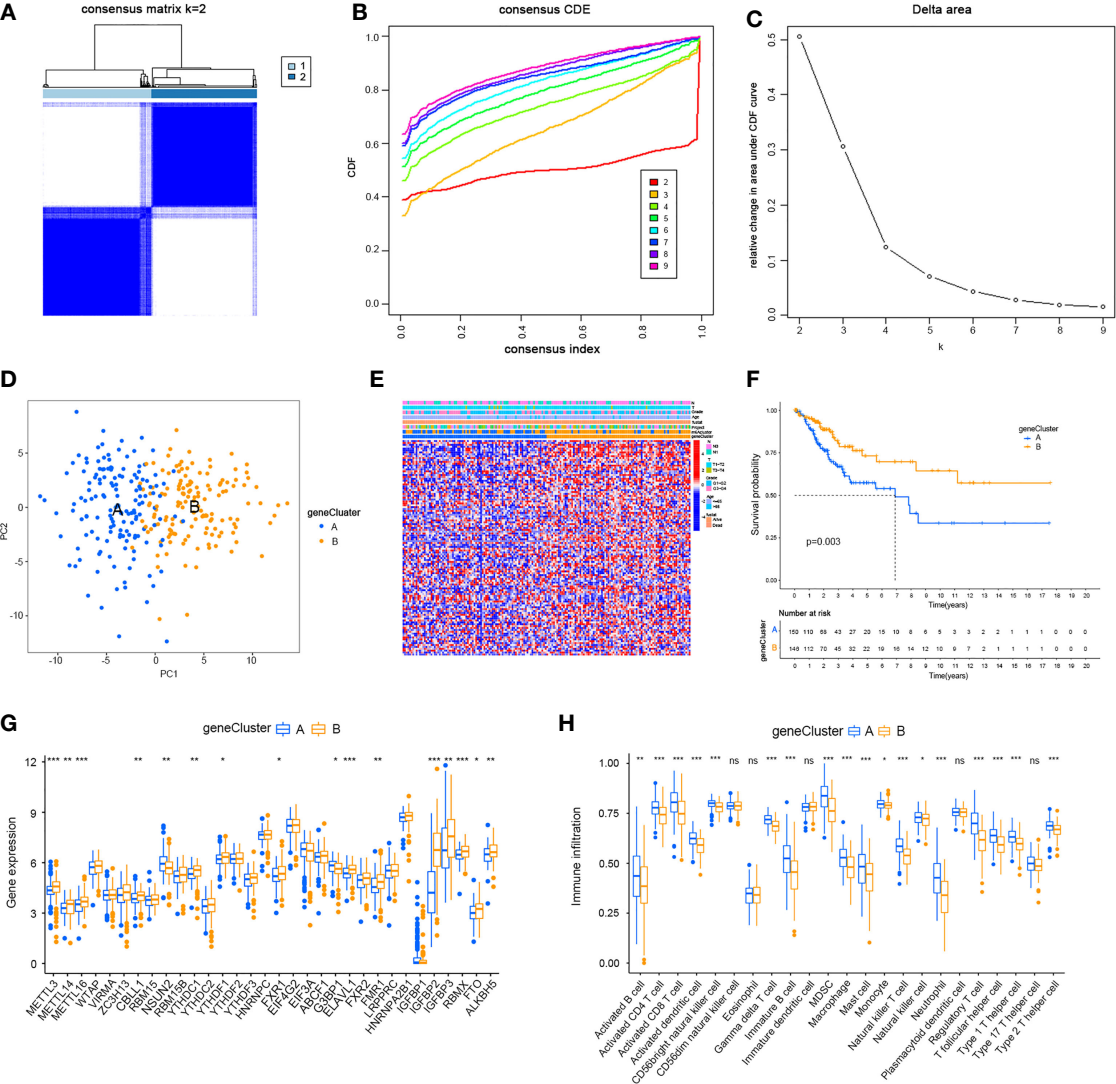
Two genomic subtypes of 114 differentially expressed genes which can affect a cancer patient’s prognosis by Unsupervised cluster analysis. **(A)** Consensus matrix. **(B)** CDF graph. **(C)** Relative change in the area under the CDF curve when k = 2–9. **(D)** Two gene clusters by Principal component analysis. **(E)** Different clinicopathological characteristics of these subgroups shown by Heat maps. **(F)** Survival analysis of m6A gene clusters by Kaplan–Meier. **(G)** Expression of m6A regulators in cervical cancer in two gene clusters. **(H)** Immune infiltration in two different m6A gene clusters in cervical cancer (*P < 0.05, **P < 0.01, and ***P < 0.001). ns, not significant.

Given the differences in the m6A regulator transcription profiles between the two m6A modification patterns, we further investigated the genetic changes and mechanisms underlying m6A modification patterns. The empirical Bayesian approach using the limma R package identified 705 differentially expressed genes (DEGs) between m6Aclusters A and B. Then, we performed functional annotation of these DEGs. Gene enrichment analysis showed significant increases in Th1-type immune response, regulation of leukocyte chemotaxis, T cell activation, G protein-coupled receptor binding and cysteine-type endopeptidase activity involved in the apoptotic signalling pathway (**Figure 4A**). KEGG enrichment analysis showed significantly increased NK cell-mediated cytotoxicity (**Figure 4B**). We further investigated the mechanism underlying these differences by unsupervised clustering analysis of DEGs. Univariate Cox regression analysis identified 114 DEGs that affected prognosis, and the cancer patients were divided into two genomic subtypes: gene cluster A (150 cases) and gene cluster B (146 cases) based on these 114 genes (**Figures 5A**–**C**). The results of gene clustering confirmed the two different m6A methylation modification patterns in cervical cancer. These two gene clusters were clearly separated (**Figure 5D**). The different clinicopathological characteristics of these subgroups are shown in **Figure 5E**. Survival analysis showed that gene cluster B was related to better survival results than gene cluster A (**Figure 5F**). Furthermore, the expression levels of all m6A regulators, with the exception of *NSUN2* and *G3BP1*, were higher in gene cluster B than in gene cluster A (**Figure 5G**). The results of ssGSEA indicated that many immune cells, including activated B cells, activated CD4^+^ T cells, activated CD8^+^ T cells, activated dendritic cells, CD56^bright^ NK cells, γδT cells, immature B cells, MDSCs, macrophages, mast cells, monocytes, NK cells, neutrophils, Treg cells, Tfh cells, Th1 cells and Th2 cells, were upregulated in gene cluster A (**Figure 5H**).

### Generation of m6A Gene Signatures and Functional Annotation

**Figure 6:**
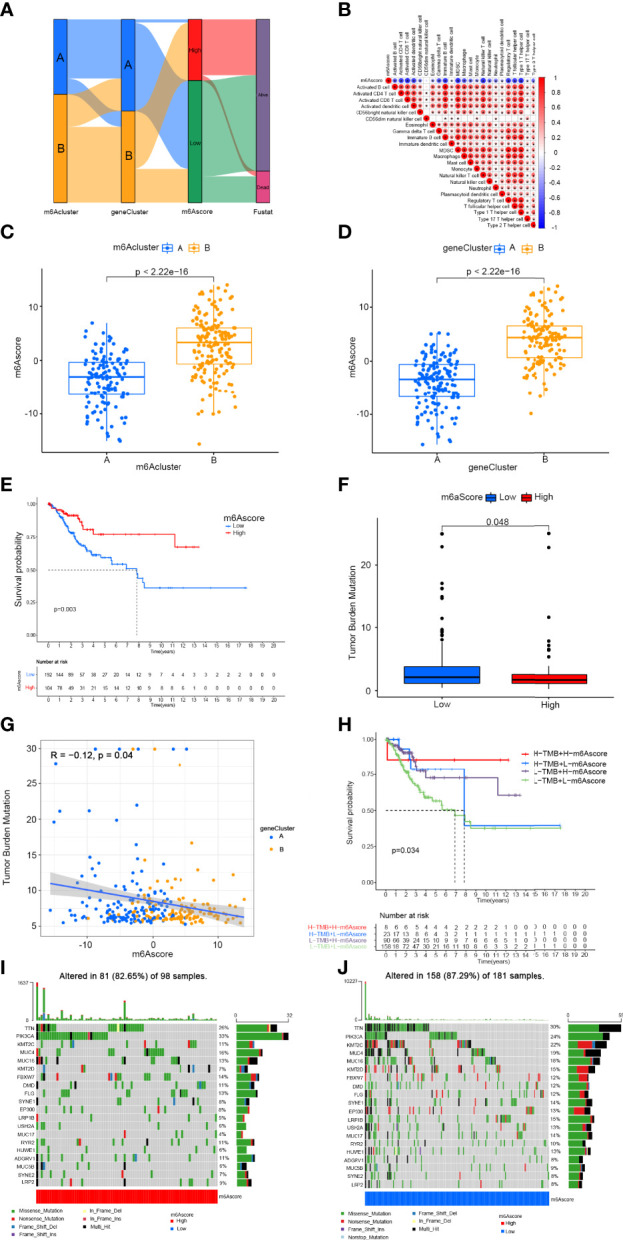
Construction of m6A gene signature and exploration of its clinical significance. **(A)** Alluvial diagram showing the changes in m6Aclusters, gene clusters, m6Ascore, and survival status. **(B)** Correlation between m6Ascore and immune cells. Blue represents negative correlation and red represents positive correlations. **(C)** Variation analysis of m6Ascore between m6Aclusters. **(D)** Variation analysis of m6Ascore between gene clusters **(E)** Survival outcomes of patients by m6Ascore. The survival outcomes of patients in the low-m6Ascore group are better. **(F)** Tumor mutation burden between different m6Ascore groups. **(G)** M6Ascore and tumour mutation burden (TMB) were negatively correlated (R = −0.12, P = 0.04). **(H)** The survival curve of patients in the subgroup of m6Ascore and TMB. **(I)** Tumor somatic mutation waterfall chart of high m6Ascores. **(J)** Tumor somatic mutation waterfall chart of low m6Ascores. Each upper bar graph shows the tumour mutation burden (TMB), and the number on the right represents the mutation frequency of each gene.

To precisely predict the m6A modification pattern at the individual patient level rather than the group level, we developed a scoring system according to the m6Ascore. We used alluvial plots to display the characteristic changes in cervical cancer patients (**Figure 6A**). As shown in the **Figure 6B**, m6Ascore was negatively related to activated CD4^+^ T cells, activated CD8^+^ T cells, activated dendritic cells, γδT cells, immature B cells, MDSCs, macrophages, mast cells, NK cells, Treg cells, Tfh cells, Th1 cells and Th2 cells. We then performed variation analysis of m6Ascore between the m6Aclusters and gene clusters. The m6Ascores of m6Acluster B and gene cluster B were markedly higher than those of m6Acluster A and gene cluster A, respectively (**Figures 6C, D**). These results indicated that the m6Ascore can be used to assess the m6A modification pattern in a specific patient sample and the TME immune cell infiltration characteristics of the tumour. We then examined prediction of patient survival outcomes based on m6Ascore. Patients with a high m6Ascore showed better survival (**Figure 6E**). Accumulating evidence suggests that the response to immunotherapy is closely related to the somatic mutations in tumour genomes. Therefore, the distributions of tumour mutation burden (TMB) in the different m6Ascore groups were analysed. The low m6Ascore group had a higher TMB than the high m6Ascore group (**Figure 6F**) and the m6Ascore was negatively associated with TMB (*R* = −0.12, *P* = 0.04) (**Figure 6G**). In addition, the high TMB group within the same m6Ascore group showed better survival (**Figure 6H**). We then analysed the differences in the distribution of somatic mutations between the high and low m6Ascore groups using the maftools package in R. The results showed that the TMB was higher in the low m6Ascore group than the high m6Ascore group (**Figures 6I**, **J**). In summary, m6Ascore classification may be related to genomic variation, and m6A modifications interact with somatic mutations.

### M6Ascore Signatures Characterised by Different Immunotherapy Landscapes

**Figure 7:**
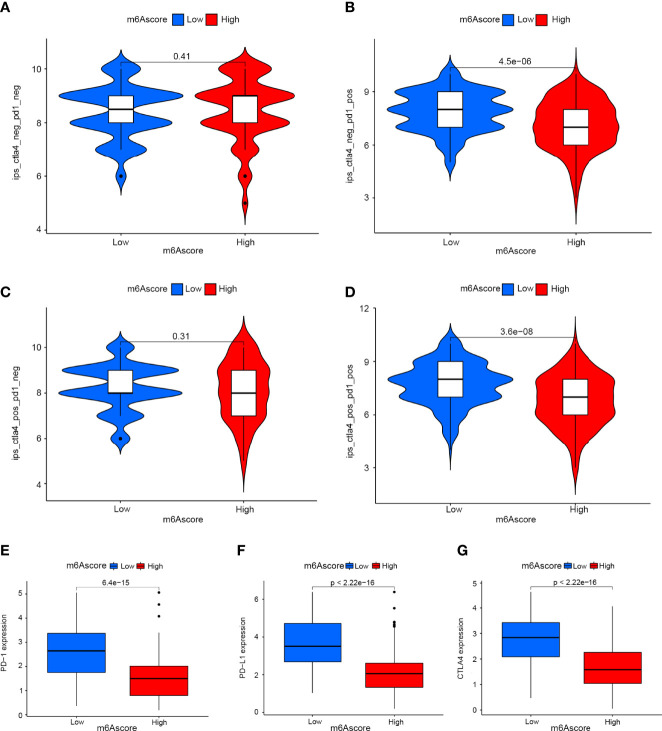
The role of m6A-scoring signature in immunotherapy. **(A–D)** The comparison of the relative distribution of immunophenoscore (IPS) between low and high m6A score groups. **(E–G)** Expression of immune checkpoints among low and high m6A score groups.

Drugs targeting PD-1 and CTLA-4 have been approved for the treatment of different types of cancer. Therefore, we explored the correlation between m6Ascore signatures and immunophenoscore (IPS) in cervical cancer patients for the purpose of predicting the response of immune checkpoint inhibitors (ICIs). We explored the differences in results of CTLA-4/PD-1 inhibitor treatment between the high and low m6Ascore groups (**Figures 7A–D**). The low m6Ascore group has higher IPS-PD-1 (**Figure 7B**) and IPS-CTLA-4/PD-1 (**Figure 7D**) scores, implying more immunogenicity on ICIs in the low m6Ascore group. Moreover, we examined the differential expression of immune checkpoint molecules between the high and low m6Ascore groups. The results indicated the low m6Ascore group has a tendency to have an immune response and respond to immunotherapy (**Figures 7E**–**G**).Taken together, these observations indicated that the m6Ascore signature is closely related to the immunotherapy response.

## Discussion

M6A is the most abundant epigenetic modification on mRNAs and long non-coding RNAs in eukaryotes (21). Reversible m6A regulators have been shown to play roles in the progression of cancer, representing a new type of post-transcriptional regulation (22). The level of m6A modification is mediated by methyltransferases (writers), demethylases (erasers) and m6A binding proteins (readers). The expression of RBM15 and HNRNPA2B1 in our study showed significant differences between cervical cancer and normal samples. Zhao et al. demonstrated that down-regulated expression of RBM15 dramatically inhibited proliferation of PAAD cell lines and RBM15 may be a prognostic biomarker and immunotherapeutic predictor in PAAD (23). Wang et al. were the first to demonstrate that m6A methyltransferase RBM15 was over-expressed in LSCC tissues and it was closely linked to the prognosis of LSCC patients (24). Guo et al. found that HNRNPA2B1 has been linked to an increased risk of ESCA by promoting the expression of fatty acid synthetic enzymes, ACLY and ACC1 (25). Yang et al. revealed that HNRNPA2B1 can activate the expression of Lin28B, thereby facilitating the malignant phenotype of ovarian cancer (26). Additionally, HNRNPA2B1 overexpression promoted tumour growth in non-small-cell lung cancers through up-regulating the expression of COX-2 and PGE2 (27). The roles of m6A methylation regulators in cervical cancer, however, are unclear. We explored methylation patterns mediated by m6A regulators and characterised TME infiltration to identify potential prognostic signatures and develop immunotherapy strategies for CESC.

In clustering analysis, we divided the CESC patients in the TCGA dataset into two m6A modification patterns based on the expression of 32 m6A regulators, designated as m6A clusters A and B. There were marked differences in TME immune cell infiltration characteristics and biological behaviour between these two m6A clusters. m6Acluster A was characterised by activation of adaptive immunity, corresponding to an immune-inflamed phenotype and m6Acluster B was characterised by the activation of innate immunity, corresponding to an immune-excluded phenotype. The immune profiles are generally divided according to the distribution of T cells (28). The immune-inflamed phenotype, named ‘hot tumour’, was characterised by the relative abundance of infiltrating immune cells. In this profile, immune cells are close to the tumour cells. The immune-excluded phenotype, named ‘cold tumour’, includes immune cells restricted to the stromal component in the tumour capsule or throughout the whole tumour, blocking the anti-tumour actions of immune cells (29). GSVA suggested that m6Acluster A was mainly enriched in inflammation and immune regulation, while m6Acluster B was mainly enriched in metabolic pathways. Tumour metabolism in the m6Acluster B inhibited tumour growth and promoted good prognosis. It has been suggested that HPVs can block the immune signalling pathways in infected keratinocytes and evade immune surveillance, contributing to the initiation and establishment of papillary immature metaplasia (30). This may be a major reason for the poor prognosis of m6Acluster A with higher levels of infiltrating immune cells.

A total of 705 DEGs between m6Acluster A and m6Acluster B, designated as the m6A-related signature genes, were identified by analysis of m6A-related gene transcription patterns. In functional analysis, these genes were shown to be closely associated with Th1-type immune response, cysteine-type endopeptidase activity involved in the apoptotic signalling pathway, T cell activation and leukocyte chemotaxis. KEGG enrichment analysis showed m6A-related genes are involved in NK cell-mediated cytotoxicity. Song et al. found that METTL3-mediated m6A methylation regulated the reaction of NK cells to IL-15 in the tumour microenvironment, thus safeguarding the steady state and immunosurveillance against tumour of NK cells (31). Ma et al. claimed YTHDF2 can control melanoma metastasis by promoting NK cells to secret perforin, granzyme B, and IFN-γ in the tumour environment (13). These observations indicated that m6A modifications shape the disparate TME landscapes. We identified two genomic subtypes based on 114 m6A signature genes that can affect prognosis in cancer patients. Furthermore, survival analysis indicated that gene cluster B was related to better survival than gene cluster A. Therefore, we propose that m6A modification may be useful in classifying the type of CESC and developing appropriate therapeutic strategies for patients. Then, in view of the individual heterogeneity of m6A modification, we developed a scoring system to evaluate the m6A modification pattern of individual CESC patients based on the m6A gene signature. Patients with a high m6Ascore showed better survival.

In addition, there were significant associations between the m6Ascore signature and immune cell infiltration. The m6Ascore was negatively associated with the abundance of activated CD4^+^ T cells, activated CD8^+^ T cells, activated dendritic cells, γδT cells, immature B cells, MDSCs, macrophages, mast cells, NK cells, Treg cells, Tfh cells, Th1 cells and Th2 cells. Studies have suggested that NK cells have a specific role in preventing HPV infection, the development and progression of cervical intraepithelial neoplasms and cancer of the uterine cervix (7). These observations were consistent with the role of T cells in immune surveillance to prevent HPV infection and consequent cancer reported previously (32).

We further explored the differences in results of CTLA-4/PD-1 inhibitor treatment between high and low m4Ascore groups. The results indicated that the levels of CTLA-4, PD-1 and PD-L1 expression were higher in CESC patients with a low m6Ascore, and the low m6Ascore group showed a better response to immunotherapy. In summary, m6A modification may be a key factor mediating the clinical response to immunotherapy and we indirectly confirmed the value of the m6Ascore in predicting outcomes of immunotherapy.

## Data Availability Statement

The datasets presented in this study can be found in online repositories. The names of the repository/repositories and accession number(s) can be found in the article/**Supplementary Material**.

## Author Contributions

WZ and PX generated and analyzed the data. JYT, RW, XW, FW, JR, SY and JAT interpreted and contributed to the draft. RH and XZ led the work. WZ and PX wrote the first draft. RH and XZ finalized the manuscript. All authors contributed to the article and approved the submitted version.

## Funding

This research was supported by the National Natural Science Foundation of China (82173554, 82101593); Natural Science Foundation of Jiangsu Province (BK20201444, BK20210844); Qing Lan Project for Excellent Young Key Teachers of Colleges and Universities of Jiangsu Province (2020); the Beijing Municipal Natural Science Foundation (7214277).

## Conflict of Interest

The authors declare that the research was conducted in the absence of any commercial or financial relationships that could be construed as a potential conflict of interest.

## Publisher’s Note

All claims expressed in this article are solely those of the authors and do not necessarily represent those of their affiliated organizations, or those of the publisher, the editors and the reviewers. Any product that may be evaluated in this article, or claim that may be made by its manufacturer, is not guaranteed or endorsed by the publisher.

## Abbreviations

CESC, Cervical Cancer; M6A, N6-methyladenosine; TCGA, The Cancer Genome Atlas; TME, Tumor microenvironments; MTC, Methyltransferase complex; NK, Natural Killer; HPV, Human papillomavirus; CTLA-4, Cytotoxic T-lymphocyte-associated protein 4; PD-1, Programmed cell death protein 1; PCA, Principal component analysis; ssGSEA, Single-sample GSEA; FPKM, fragments per kilobase of transcript per million mapped reads; TPM, transcripts per kilobase million; CNV, copy number variants;DEGs, differentially expressed genes; TMB, tumour mutation burden; IPS, immunophenoscore; CIN, cervical intraepithelial neoplasms; ICIs, immune checkpoint inhibitors.
