# Supplementary material for: m6A Regulator-Mediated Tumour Infiltration and Methylation Modification in Cervical Cancer Microenvironment
Source: Front Immunol. 2022 Apr 29;13:888650. doi: 10.3389/fimmu.2022.888650 (PMC9098799; doi:10.3389/fimmu.2022.888650)
Supplement: Supplementary file 1 [file DataSheet_1.docx]

**Supplementary Table 1**

32 m6A regulators related to the survival condition of patients with cervical cancer

| **ID** | **HR** | **HR.95L** | **HR.95H** | **P-value** | **km** |
| --- | --- | --- | --- | --- | --- |
| *METTL3* | 1.263 | 0.828 | 1.925 | 0.279 | 0.092 |
| *METTL14* | 1.233 | 0.760 | 2.001 | 0.397 | 0.038 |
| *METTL16* | 0.718 | 0.434 | 1.189 | 0.198 | 0.030 |
| *WTAP* | 1.411 | 0.862 | 2.310 | 0.171 | 0.042 |
| *WTAP* | 1.528 | 0.962 | 2.428 | 0.073 | 0.012 |
| *ZC3H13* | 1.502 | 1.048 | 2.153 | 0.027 | 0.005 |
| *CBLL1* | 1.310 | 0.774 | 2.217 | 0.314 | 0.112 |
| *RBM15* | 1.143 | 0.647 | 2.019 | 0.646 | 0.084 |
| *NSUN2* | 1.153 | 0.830 | 1.603 | 0.395 | 0.045 |
| *RBM15B* | 1.200 | 0.775 | 1.860 | 0.413 | 0.154 |
| *YTHDC1* | 1.078 | 0.691 | 1.681 | 0.740 | 0.091 |
| *YTHDC2* | 0.998 | 0.683 | 1.457 | 0.990 | 0.351 |
| *YTHDF1* | 0.863 | 0.522 | 1.426 | 0.564 | 0.105 |
| *YTHDF2* | 1.465 | 0.818 | 2.621 | 0.199 | 0.005 |
| *YTHDF3* | 1.507 | 0.936 | 2.426 | 0.091 | 0.007 |
| *HNRNPC* | 1.613 | 0.852 | 3.054 | 0.142 | 0.009 |
| *FXR1* | 1.143 | 0.778 | 1.681 | 0.496 | 0.026 |
| *EIF4G2* | 1.243 | 0.825 | 1.874 | 0.299 | 0.118 |
| *EIF3A* | 1.356 | 0.966 | 1.904 | 0.078 | 0.030 |
| *ABCF1* | 1.149 | 0.731 | 1.806 | 0.546 | 0.062 |
| *G3BP1* | 1.882 | 1.073 | 3.302 | 0.028 | 0.013 |
| *ELAVL1* | 0.795 | 0.452 | 1.398 | 0.426 | 0.038 |
| *FXR2* | 0.983 | 0.623 | 1.551 | 0.942 | 0.082 |
| *FMR1* | 0.738 | 0.529 | 1.029 | 0.074 | 0.021 |
| *LRPPRC* | 1.465 | 0.937 | 2.290 | 0.094 | 0.023 |
| *HNRNPA2B1* | 1.657 | 0.872 | 3.147 | 0.123 | 0.035 |
| *IGFBP1* | 1.199 | 0.860 | 1.671 | 0.285 | 0.020 |
| *IGFBP2* | 0.937 | 0.832 | 1.056 | 0.288 | 0.052 |
| *IGFBP3* | 1.009 | 0.879 | 1.158 | 0.900 | 0.115 |
| *RBMX* | 0.907 | 0.521 | 1.578 | 0.729 | 0.016 |
| *FTO* | 1.011 | 0.693 | 1.474 | 0.955 | 0.062 |
| *ALKBH5* | 0.993 | 0.639 | 1.545 | 0.976 | 0.097 |
